# Longitudinal analysis of XEN45 gel stent bleb morphology using bleb grading scales, anterior segment-OCT, in vivo confocal microscopy, and impression cytology

**DOI:** 10.1007/s00417-025-06952-0

**Published:** 2025-10-03

**Authors:** Néstor Ventura-Abreu, Blanca Molins, José Guerra-Meniconi, Sara Labay-Tejado, Sofía Porto, María Jesús Muniesa, Elena Millà, Marta Pazos

**Affiliations:** 1 Institut Clínic d’Oftalmologia. Hospital Clínic de Barcelona, Universitat de Barcelona, Barcelona, Spain; 2 Fundació de Recerca Clínic Barcelona-Institut d’Investigacions Biomèdiques August Pi i Sunyer, Barcelona, Spain; 3 Hospital Sagrat Cor, Barcelona, Spain

**Keywords:** Glaucoma surgery, Open-angle glaucoma, Bleb-forming devices, In vivo confocal microscopy, Anterior-segment optical coherence tomography, Impression cytology

## Abstract

**Purpose:**

To characterize bleb morphology and conjunctival features after XEN45 Gel Stent surgery (XGS) using slit-lamp bleb grading, anterior segment optical coherence tomography (AS-OCT), in vivo confocal microscopy (IVCM), and impression cytology analysis (ICA), in both success and failure cases.

**Methods:**

Twenty-four eyes (24 patients) underwent XGS, with 20 included in the final analysis. In addition to a full ophthalmic exam, blebs were evaluated using Moorfield’s Bleb Grading System (BGS), AS-OCT, IVCM, and ICA. AS-OCT metrics included bleb-wall and subepithelial cyst-like structure area and density (BECSA/BECSD, BSCSA/BSCSD), bleb height (BH), bleb thickness (BT), and epithelial thickness (BET). IVCM parameters included stromal meshwork reflectivity (SMR), goblet cell density (GCD), epithelial microcyst density and area (EMD, EMA), and dendritic cell density (DCD). ICA assessed MUC5AC and HLA-DR/cell area ratio. Success was defined as an IOP < 21 mmHg and ≥ 20% reduction at six months. Imaging parameters were correlated with surgical outcomes.

**Results:**

At six months, 11 (55%) cases were classified as successful and 9 (45%) as failures. BGS showed significantly larger blebs in the success group (88.9% with maximal diffusion area grade ≥ 3 vs. 22.1%, *p* < 0.05). AS-OCT detected persistent epithelial and subepithelial changes in successful blebs, with significantly higher BECSA (0.11 vs. 0.02 mm², *p* < 0.02). Preoperatively, successful cases showed higher GCD on IVCM (67.3 vs. 39.2 cells/mm², *p* = 0.25) and mucin expression on ICA (MUC5AC/cell ratio: 1.28 vs. 0.01, *p* = 0.11). Postoperative IVCM also showed trends toward increased EMD in both groups, though not statistically significant.

**Conclusion:**

Successful XGS blebs were associated with higher preoperative goblet cell density (IVCM) and mucin expression (ICA). Postoperatively, AS-OCT parameters—especially epithelial and subepithelial changes—showed the strongest correlation with IOP, highlighting AS-OCT as the most clinically applicable tool for monitoring bleb function.

**Trial registration:**

ClinicalTrials.gov Identifier NCT04727411, January 22, 2021.

**Supplementary Information:**

The online version contains supplementary material available at 10.1007/s00417-025-06952-0.

## Key messages

*****What was known before***:**

- Bleb-forming devices (such as XEN45 Gel Stent, XGS) have been characterized as flatter, less extensive blebs compared to other filtering procedures (trabeculectomy, deep sclerectomy)
- The characteristics of functioning XGS blebs have been scarcely described, and the preoperative characteristics and changes during the bleb maturation that might conform to a successful bleb have not been detailed.

*****What this study adds***:**

- The conjunctival bleb characteristics of the successful cases resemble those of the traditional glaucoma
- Incorporating bleb-wall epithelium cyst-like structure area measurements (BECSA) with anterior-segment optical coherence tomography or mucin level expression with impression cytology analysis could help to differentiate between functioning and failed blebs instead of solely relying on clinical evaluation

**Supplementary Information:**

The online version contains supplementary material available at 10.1007/s00417-025-06952-0.

## Introduction

Glaucoma surgery plays a crucial role in managing this progressive disease, with trabeculectomy widely recognized as the gold-standard procedure. Over time, numerous implants and techniques have been developed to achieve a balance between safety and effectiveness, despite the proven efficacy of trabeculectomy. Among these advancements, minimally invasive glaucoma surgeries (MIGS) have been designed to enhance the physiological outflow pathways while preserving other ocular tissues [1]. Despite these intentions, MIGS have demonstrated only moderate, mid-term efficacy in most cases [2]. Another alternative includes bleb-forming devices, which may not be as effective as trabeculectomy [3] but provide a considerable intraocular pressure (IOP) reduction and a favorable safety profile due to their design.

One of the most widely implanted bleb-forming devices is the XEN45 Gel Stent (XGS) (Abbvie^®^, Irvine, CA, USA). The 6 mm-long shunt, with a 45 μm lumen, is made of biocompatible gelatine and works by redirecting aqueous humor to the subconjunctival space, creating a filtering bleb that differs from the conventional blebs observed after a trabeculectomy [4]. 

Proper bleb evaluation is critical in a trabeculectomy to identify those at risk of failure, even when IOP values are within the normal range [5]. Grading scales based on slit-lamp examination provide a reliable method for assessing blebs [6], but other approaches have helped to better understand aqueous humor drainage mechanisms [7, 8]. 

However, only a few studies have prospectively evaluated the bleb morphology of the XGS using various imaging techniques. The purpose of the present study is to investigate the evolution of the XGS bleb features over time using four different methods: bleb grading scale (BGS), anterior segment optical coherence tomography (AS-OCT), in vivo confocal microscopy (IVCM), and impression cytology analysis (ICA), and a potential association with surgical success.

## Methods

This prospective, observational, single-center study consecutively included patients with a surgical indication of XGS. Eligible patients had mild-to-moderate open-angle glaucoma [9] (including pseudoexfoliation or pigmentary dispersion syndrome) and no signs of glaucoma progression; with over-target IOP, or well-controlled, target-IOP cases, but aiming for a lower IOP with fewer glaucoma medications (i.e., for medical therapy intolerance). If the patient had a significant concomitant cataract, combined phaco-XGS was also allowed. The exclusion criteria were: severe or end-stage glaucoma (following Mills’ classification); any form of angle closure (Shaffer 2 or below) and other types of secondary glaucoma; and patients with prior ocular surgery, excluding selective laser trabeculoplasty or uncomplicated phacoemulsification 6 months before the inclusion in the study.

This study was conducted under the principles of the Declaration of Helsinki. Approval was obtained from the Ethics Committee of Hospital Clinic of Barcelona (HCB/2019/0035). Informed consent was secured before inclusion. The study was registered in the National Library of Medicine (clinicaltrials.gov, NCT04727411). All surgeries were carried out between January 2021 and October 2022 by the same glaucoma surgeon (MP).

In the preoperative visit, all patients had a complete ophthalmologic examination including IOP measured by Goldmann applanation tonometry, visual acuity in decimal scale (Snellen Chart), slit-lamp examination, gonioscopy, optic nerve evaluation, endothelial cell count and Humphrey visual field testing, and optic nerve and macula OCTs (Carl Zeiss Meditec Inc., Dublin, CA). After the surgery, the patients were reevaluated on days 1, weeks 1 and 2, and months 1, 3, and 6. Any other visits apart from the original schedule were indicated at the surgeon’s discretion. During both the preoperative and corresponding postoperative visits, bleb images were acquired sequentially using the slit-lamp camera, anterior segment optical coherence tomography (AS-OCT), In Vivo Confocal Microscopy (IVCM), and lastly, the Impression Cytology Analysis (ICA) 30 min after IVCM.

### Surgical technique and postoperative care

The standard ab-interno closed conjunctiva surgical technique of the XGS implantation is described elsewhere [10]. Before implantation, 4% lidocaine was injected in the superior-nasal conjunctiva, and an ab-interno subconjunctival stent was placed through an inferior-temporal clear corneal incision to exit at the subconjunctival space at 3 mm from the limbus. In all cases, free mobility of the implant was verified at the end of the procedure, and 0.1 ml of mitomycin C (MMC) at 0.1 mg/ml was injected posteriorly. In patients who underwent combined phaco-XGS surgery, cataract extraction and in-the-bag intraocular lens placement were performed before XEN implantation. No primary needling or *ab externo* implantation were performed.

Topical ofloxacin q.i.d. was administered during the first week, and dexamethasone 0.1% drops six times per day and a steroid ointment overnight were indicated during the first month and tapered during the following two months.

### Clinical outcomes

IOP and the number of hypotensive medications were registered preoperatively and during the follow-up. Clinical and bleb analysis were conducted on the two study groups: success and failure. According to the definitions provided by the European Glaucoma Society Guide on Surgical Innovation for glaucoma [1], success was defined as achieving a final IOP below 21 mmHg along with a ≥ 20% reduction from baseline (mild glaucoma). Failure was defined as not reaching the target IOP, an IOP below 6 mmHg with shallow anterior chamber, choroidal detachments or hypotony maculopathy, further glaucoma surgery, and/or sight-threatening complications. Postoperative complications were also reported as early or late (3-month cutoff).

### Bleb grading scale (BGS)

Slit lamp pictures using a DC-4 digital camera mounted in the slit lamp (Topcon Corporation, Tokyo, Japan) were taken to obtain filtering bleb photography images at baseline and months 1, 3, and 6 after surgery. A blinded examiner (JGM) classified the photographs at every postoperative visit using Moorfield’s Bleb Grading System [11]. Bleb grading system parameters measured were the following: central and maximal bleb areas (1–5), bleb height (1–4), central bleb, peripheral bleb and non-bleb vascularity (1–5), and subconjunctival blood (0–1).

### AS-OCT

The bleb morphology was also assessed with AS-OCT (anterior module segment, DRI-OCT Triton, Topcon Corporation, Tokyo, Japan). A radial anterior segment 6 mm protocol was used to get images from the entire bleb. An external fixation point was placed to examine the bleb in the right or left downgaze, correspondingly; then, the central point of the cart-wheel scan was in the middle of the subconjunctival trajectory of the XGS. The scans were obtained at months 1, 3 and 6. The images were analyzed in two ways: a morphologic classification described by Lenzhofer et al. [4] [uniform bleb (no fluid-filled hyporeflective spaces in subconjunctival space) (1), subconjunctival separation (multiple small spaces in more superficial layers) (2), microcystic multiform (3), and multiple internal layers (4)]; and a quantitative analysis including the parameters defined by Singh et al. (bleb height, BH; bleb epithelial thickness, BET; and bleb wall thickness, BT, all in µm) and the four described by Sacchi et al. [12] (bleb-wall epithelium cyst-like structure density, BECSD; and bleb-wall sub-epithelium cyst-like structure density, BSCSD, both in microcysts/image); and the corresponding areas (BECSA and BSCSA, in µm^2^). The AS-OCT measurements were crafted using the ImageJ software (provided in the public domain by the National Institutes of Health, Bethesda, MD, USA; https://imagej.nih.gov/ij/)*.*

### IVCM

The IVCM analysis was performed with the laser scanning confocal microscope (HRT III Rostock Cornea Module, Heidelberg Engineering, Heidelberg, Germany). Except for the eye position (right or left downgaze for the right or left eye accordingly), the appearance of the dendritic cells, goblet cells, and epithelial microcysts, and the examination protocol are described elsewhere. [13] The ImageJ software was used as well for the IVCM measurements. A custom-made macro was used for the microcyst area and stromal reflectivity, using manual measurements only in dubious cases. Measurements were reported in cells/mm^2^ (dendritic cell density, DCD; goblet cell density, GCD); cysts/mm^2^ (epithelial microcyst density, EMD); µm^2^ (epithelial microcyst area, EMA) and an arbitrary scale for the stromal meshwork reflectivity (SMR), all previously reported by Mastropasqua et al. [13].

### ICA

The impression cytology sample collection was performed 30 min after the last examination. After topical anesthesia, the sample was obtained using the Eyeprim device (OPIA Technologies, Paris, France). It consists of a preloaded 69 mm^2^ membrane adapted to the curvature of the eyeball. The membrane was placed on the conjunctiva, 2–3 mm posterior to the limbus where the subconjunctival trajectory of XGS started, for 5 s and gently retired. The membrane was expelled further by pressing the button until it was detached from the device. Within 15 min the membrane was immersed in 0.5% paraformaldehyde for 10 min and then washed twice with phosphate-buffered saline (PBS). Cells were then permeabilized with Triton X-100 for five minutes, washed twice with PBS, and blocked with 3% filtered BSA for 1 h. Afterward, conjugated antibodies from Abcam (HLADR-AF647 1:500, MUC5AC-AF555 1:100, cytokeratin 19-AF488 1:100) were added and incubated at 4 C overnight. Then, samples were washed twice with PBS, and nuclei were counterstained with 4′,6-diamidino-2-phenylindole (DAPI). Controls were stained with secondary antibodies only. Stained cells were washed and covered with Prolong Gold antifade reagent (Life Technologies). Images of immunostained cells were recorded on the high-speed spectral confocal microscope Leica TCS-SP5 and analyzed with ImageJ software. A minimum of 5 images per sample were acquired. A semi-automated macro was developed to determine the area covered by cells, based on DAPI and cytokeratin 19 staining. Mucin and HLADR levels were expressed as the area covered by MUC5AC staining and the area covered by HLADR staining divided by the area occupied by cells, respectively.

### Statistical analysis

Data were analyzed using Stata v14.1 (StataCorp, College Station, TX, USA). Based on previous literature that identified significant differences between the success and failure groups [14] and assuming an 80% power with a two-sided significance level of 5%, we estimated a minimum sample size of 20 cases using the reported success rates.

The Shapiro-Wilk test was used to assess sample distribution. Intra and intergroup comparisons were performed using the Wilcoxon signed-rank test and the Mann-Whitney U-test. Repeated measures analysis of variance (ANOVA) with Greenhouse-Geisser correction was carried out to compare variables over time. Fisher’s exact test (with Bonferroni’s correction) was applied to analyze the proportions for MBGS and AS-OCT classifications between success and failure groups. The agreement between each imaging variable and intraocular pressure (IOP) at six months was assessed using Spearman’s correlation. A two-tailed *p*-value of < 0.05 was considered statistically significant.

## Results

The study included 24 eyes from 24 patients. One was excluded from the analysis due to improper XGS placement, and three other cases discontinued postoperative follow-up visits after the first (two cases) and third (one case) months, for unknown reasons.

The baseline characteristics of the remaining cases (*n* = 20) are summarized in Table 1. There were similarities between the success and failure groups.

**Table 1:** Demographic and ocular characteristics of the subjects included in the study and their baseline characteristics

Demographics/ocular characteristics	Overall group (n=20)*	Success group (n= 11)**	Failure group (n=9)***	*p* value
Age (in years), mean (SD)	75.4 (8.9)	74.7 (9.0)	76.2 (9.3)	0.56^Ω^
Gender, n (%) Female	11 (55.0)	8 (72.7)	3 (33.3)	0.18^&^
Race				1.00^&^
- White	19 (95.0)	10 (90.9)	9 (100.0)	
- Hispanic/latino	1 (5.0)	1 (9.1)	0 (0)	
Glaucoma type				0.341^&^
- Primary open angle glaucoma	13 (65.0)	6 (54.6)	7 (77.8)	
- Pseudoexfoliative glaucoma	4 (20.0)	2 (18.2)	2 (22.2)	
- Steroid-aggravated glaucoma^#^	3 (15.0)	3 (27.3)	0 (0.0)	
Type of procedure				
- Phaco-XGS	15 (75.0)	9 (81.8)	6 (66.7)	0.617^&^
- Standalone XGS	5 (25.0)	2 (18.2)	3 (33.3)	
Postoperative maneuvers				0.653^&^
- Needling	9 (55.0)	4 (36.4)	5 (55.6)	
- No needling	11 (45.0)	7 (63.6)	4 (44.4)	
Baseline characteristics, mean (SD)				
- BCVA (in Snellen)	0.7 (0.2)	0.7 (0.2)	0.7 (0.2)	0.91^Ω^
- Visual field (MD in dB)	-4.48 (4.3)	-5.9 (5.2)	-3.0 (2.8)	0.31^Ω^
- IOP (mmHg)	19.6 (4.0)	20.7 (3.9)	18.1 (3.9)	0.19^Ω^
- Glaucoma medications^$^	3 (2-3)	3 (2-3)	3 (2-3)	0.97^Ω^
- Glaucoma status^$$^				
o Controlled	15 (75.0%)	8 (72.7)	7 (77.8)	1.00^&^
o Uncontrolled	5 (25.0%)	3 (27.3)	2 (22.2)	
- ECD (cells/mm^2^)	1968 (583)	2112 (456)	1791 (695)	0.27^Ω^
- pRNFL (µm)	72.0 (10.6)	69.8 (12.0)	74.6 (8.6)	0.29^Ω^
- Diabetes mellitus				0.64^&^
o Yes	5 (25.0)	3 (27.3)	2 (22.2)	
o No	15 (75.0)	8 (72.7)	7 (77.8)	

### Intraocular pressure and number of medications

Overall, the mean IOP decreased significantly from 19.6 ± 4 mmHg at baseline with a mean of 2.6 medications to 16.2 ± 4.9 mmHg with 0.6 medication at six months (Supplementary Material (SM) 1) (*p* < 0.001, ANOVA test). By the end of the study, 11 eyes (55%) met the predefined success criteria (success group), while 9 eyes (45%) did not (failure group). Two patients discontinued follow-up after the third postoperative visit.

As expected, IOP and glaucoma medications were significantly lower in the success group (*p* < 0.001. In the failure group, only the reduction in glaucoma medication was significant (SM 1).

### Visual acuity, VF parameters, and structural outcomes

Visual acuity (Snellen scale) improved slightly (from 0,7 to 0,8, *p* = 0.03), as did the peripapillary retinal nerve fiber layer (pRNFL) thickness (from 72.0 to 77.6 μm, *p* < 0.05). Those changes were likely attributed to cataract extraction [15]. 

Visual field remained stable over the postoperative period, from a preoperative mean deviation of −4.48 to −5.14 decibels. See more details for these variables in SM2.

### Complications and postoperative interventions

There were two cases with intraoperative complications. In one eye, a subconjunctival hemorrhage obstructed visualization, leading to an accidental suprachoroidal placement of the implant (this eye was excluded from the study as mentioned above). In the other case, severe floppy iris syndrome occurred during phacoemulsification but resolved without further incidents.

Postoperative sight-threatening complications, both early and late, were rare. Four patients developed self-limited numerical hypotony during the first postoperative week, which resolved either spontaneously or with topical treatment. One eye developed cystoid macular edema at 3 months, successfully treated with topical medication. However, bleb fibrosis (identified based on clinical appearance and elevated IOP) occurred in nine eyes (42.8%), all of which required bleb needling with 5-fluorouracil. Of these, two cases occurred within the first month, and seven during the second and third months. The proportion of patients requiring secondary needling was similar between the success/failure groups (36.4 vs. 50.0%; *p* = 0.66, Fisher’s exact test).

The case with the misplaced XGS required a trabeculectomy to further control IOP; the rest of the patients did not require a subsequent glaucoma filtering surgery during the study follow-up.

### Slit lamp bleb grading

At six months, the proportion of surgeries achieving a bleb area grade 3 or higher (> 50%) was significantly greater in the success group, specifically for the maximal diffusion area (88.9 vs. 22.1%, *p* < 0.05). Bleb height was not higher than grade 2 for any case; in fact, the proportion of flat blebs (grade 1) was similar between groups (87.5 vs. 55.6%, *p* = 0.29). Interestingly, bleb hyperemia (grade 3 or higher) was slightly more prevalent in the success group compared to the failure group (44.4 vs. 25.0, *p* = 0.62).

### AS-OCT

In qualitative analysis, type 2 blebs (subconjunctival separation) were most frequently observed in the success group (50.0%, 44.4%, and 44.40% at the 1, 3, and 6 months, respectively). In contrast, although type 2 blebs were initially comparable between groups (5 vs. 3 cases at 1 month), type 1 (uniform bleb) predominated in the failure group by the end of follow-up (6 vs. 2 cases). There was no statistically significant association between the bleb morphology and the success/failure outcome (*p* = 0.08, Fishers’ exact test).

Table 2 summarizes quantitative AS-OCT measurements during follow-up. Overall, most parameters decreased over time. However, subgroup analysis revealed that in the success group, both BH and BECSD remained stable over time, whereas these measures decreased in the failure group. This trend extended to the epithelial structure area, with BESCA significantly lower in the failure group (22547.9 µm^2^) compared to the success group (108768.9 µm^2^; *p* = 0.02).

**Table 2:** Anterior segment OCT (AS-OCT) bleb quantitative measurements over time

	BECSD			BECSA			BSCSD			BSCSA		
	M1	M3	M6	M1	M3	M6	M1	M3	M6	M1	M3	M6
Overall	8.2 (5.5)	5.5 (4.3)	5.6 (4.5)	41321.8 (36696.5)	33115.9 (36125.6)	65658.4 (93729.6)	4.7 (4.4)	3.9 (4.2)	1.9 (2.7)	176599.5 (277716.2)	251195.8 (476213.2)	155215.4 (354701.1)
Success	8.2 (6.5)	6.9(4.8)	7.1(4.5)	35752.4 (32506.5)	43870.0 (39704.7)	108768.9 (114547.2)	4.4 (3.6)	2.4 (3.4)	1.3 (2.2)	161239.5 (155677.0)	415114.6 (637408.5)	278768.4 (480479.5)
Failure	8.1 (4.5)	4.1(3.6)	4.0(4.3)	48128.7 (42221.4)	22361.8 (30603.5)	22547.9 (36906.7)	5.0 (5.4)	5.3 (4.6)	2.4 (3.1)	193666.2 (381637.0)	87277.1 (123124.8)	31662.5 (46311.8)
*p* value*	1.00	0.18	0.13	0.47	0.16	**0.02**	0.97	0.13	0.46	0.74	0.96	0.68
	SMR			GCD			EMD					
	M1	M3	M6	M1	M3	M6	M1	M3	M6			
Overall	562.7 (167.5)	541.4 (210.8)	519.1 (246.7)	230.5 (86.3)	212.5 (83.0)	210.1 (78.1)	78.8 (21.9)	72.2 (17.2)	71.5 (23.2)			
Success	568.9 (154.8)	615.5 (258.5)	610.7 (322.0)	259.4 (105.2)	227.0 (100.1)	238.9 (98.4)	83.5 (23.4	70.9 (17.0)	79.9 (25.5)			
Failure	555.8 (190.0)	467.4 (123.7)	427.4 (82.3)	198.5 (46.1)	198.1 (64.2)	181.2 (37.5)	73.7 (20.1	73.6 (18.3)	64.2 (119.4)			
*p* value*	0.74	0.23	0.31	0.14	0.63	0.35	0.22	0.89	0.17			

*BECSA* bleb-wall epithelium cyst-like structure area (µm^2^), *BECSD* bleb-wall epithelium cyst-like structure density (microcysts/image), *BSCSA* bleb-wall sub-epithelium cyst-like structure area(µm^2^), *BSCSD* bleb-wall sub-epithelium cyst-like structure density (microcysts/image), *BH* Bleb Height (µm), *BT* Bleb Thickness (µm), *BET* Bleb Epithelial Thickness (µm); * Mann-Whitney U-test (success-failure comparison)

### IVCM

Baseline and postoperative IVCM parameters are detailed in Table 3. Goblet cells (GC) were observed as large, hyperreflective, and oval-shaped cells with hyporreflective nuclei, larger than the surrounding epithelial cells [16, 17].

**Table 3:** In vivo confocal microscopy (IVCM) study findings during the follow-up

	SMR				GCD				EMD			
Mean (SD)	Preop	M3	M6	*p* value **	Preop	M3	M6	*p* value **	Preop	M3	M6	*p* value **
Overall	96.0 (27.9)	89.8 (17.9)	89.4 (15.2)	0.43	55.2 (45.6)	53.8 (43.4)	48.9 (27.7)	0.86	21.6 (12.3)	36.2 (19.9)	30.7 (18.3)	0.03^#^
Success	98.0 (35.0)	89.5 (18.3)	93.6 (14.8)	0.76	67.3 (54.1)	57.5 (53.3)	49.5 (28.8)	0.78	24.9 (13.4)	45.2 (17.0)	30.5 (20.5)	0.06
Failure	93.4 (15.7)	90.1 (18.7)	84.7 (15.0)	0.41	39.2 (25.8)	51.2 (33.9)	48.2 (28.3)	0.43	17.3 (9.7)	27.1 (19.2)	30.9 (16.7)	0.19
*p* value*	0.17	0.76	0.46		0.25	0.92	0.87		0.14	**0.03**	0.81	
	EMA				DCD							
Mean (SD)	Preop	M3	M6	*p* value **	Preop	M3	M6	*p* value **				
Overall	7281.0 (6087.7)	25976.2 (30819.9)	20228.6 (17858.1)	0.01^##^	15.3 (14.4)	24.9 (22.2)	22.5 (18.1)	0.26				
Success	9216.7 (7064.2)	28433.3 (33810.1)	17791.7 (19984.5)	0.05^**^	17.9 (12.3)	24.1 (17.7)	23.1 (21.7)	0.80				
Failure	4701.2 (3302.8)	22694.5 (27967.2)	23482.7 (15070.8)	0.11^**^	12.1 (16.9)	25.6 (27.1)	21.9 (14.3)	0.25				
*p* value*	**0.04**	0.83	0.23		0.15	0.79	0.80					

3G. * Mann-Whitney U-test, comparing success vs failure cases. *DCD* dendritic cell density (cells/mm^2^), *EMD* epithelial microcyst density (microcysts/mm^2^), *EMA* epithelial microcyst area (µm^2^), *GCD* goblet cell density (cells/mm^2^), *M3* month3, *M6* month 6, *SMR* stromal meshwork reflectivity (arbitrary scale). **ANOVA for repeated measures. ^#^ EMD was significantly higher when comparing the preoperative and the 3-month visit. ^##^ EMA was significantly higher in both postoperative visits

**Fig. 1:**
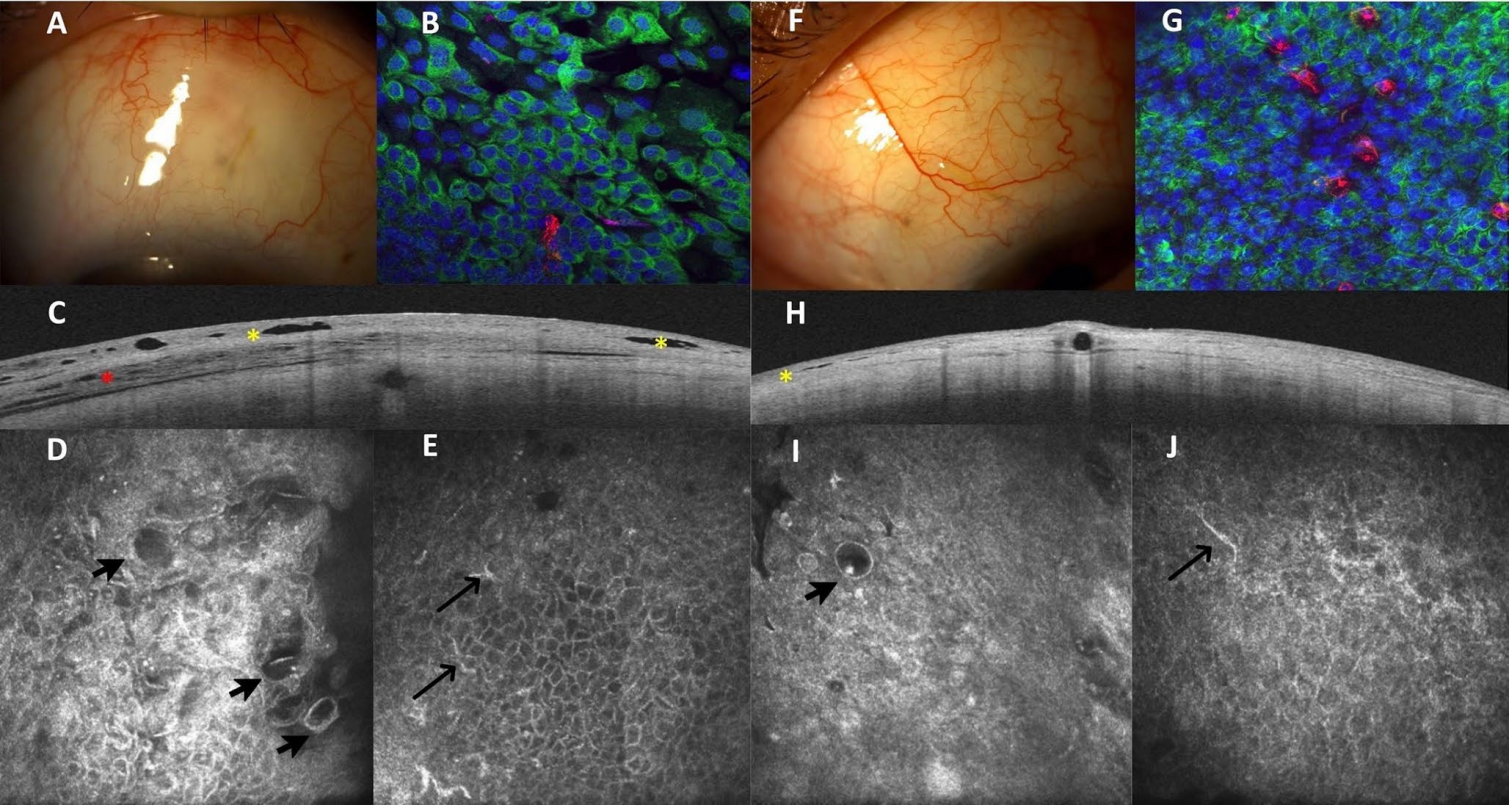
Six-month postoperative appearance of successful (**A**-**E**) and failed (**F**-**J**) cases. **A**, **F**: slit lamp pictures of the bleb. **B**, **G**: ICA confocal images. The epithelial cells of the conjunctiva (nuclei and surrounding cytokeratin stained in blue and green, respectively) adopt a looser arrangement than the failed blebs. Mucin levels were lower after 6 months (red staining) in the failure group. **C**-**H**: AS-OCT pictures. The yellow asterisk indicates the bleb-wall epithelium cyst-like structures, and the red asterisk shows the bleb-wall subepithelium cyst-like structure, only visible in the successful case. **D**, **E**, **I**, **J**: IVCM images of the conjunctiva. **D**, **I** show the large, epithelial microcysts (arrowheads), more numerous and clustered in the successful surgeries. Other areas of the bleb showed more epithelial cells with dendritic cells (**E**, **J**, black arrows)

Microcysts, on the contrary, appeared as round-to-oval structures, with hyperreflective walls and optically clear content, occasionally containing amorphous material or mononucleate hyperreflective components (Fig. 1).

The baseline-postoperative comparisons showed a slight reduction in GC count in both groups. At baseline and postoperatively, GCs were sparsely distributed. While GCs were more abundant in the success group at baseline, the differences were not statistically significant.

Epithelial microcyst density and area increased significantly postoperatively (*p* < 0.05). Preoperatively, EMA was significantly higher in the success group (*p* = 0.04). Three months after surgery, successful blebs had significantly higher EMD values (*p* = 0.03). In the sixth month, these parameters had tripled compared to baseline.

Stromal reflectivity (SMR) decreased over time but remained slightly higher in the success group, though the difference was not statistically significant. Dendritic cell counts showed no significant changes during follow-up.

### ICA

**Fig. 2:**
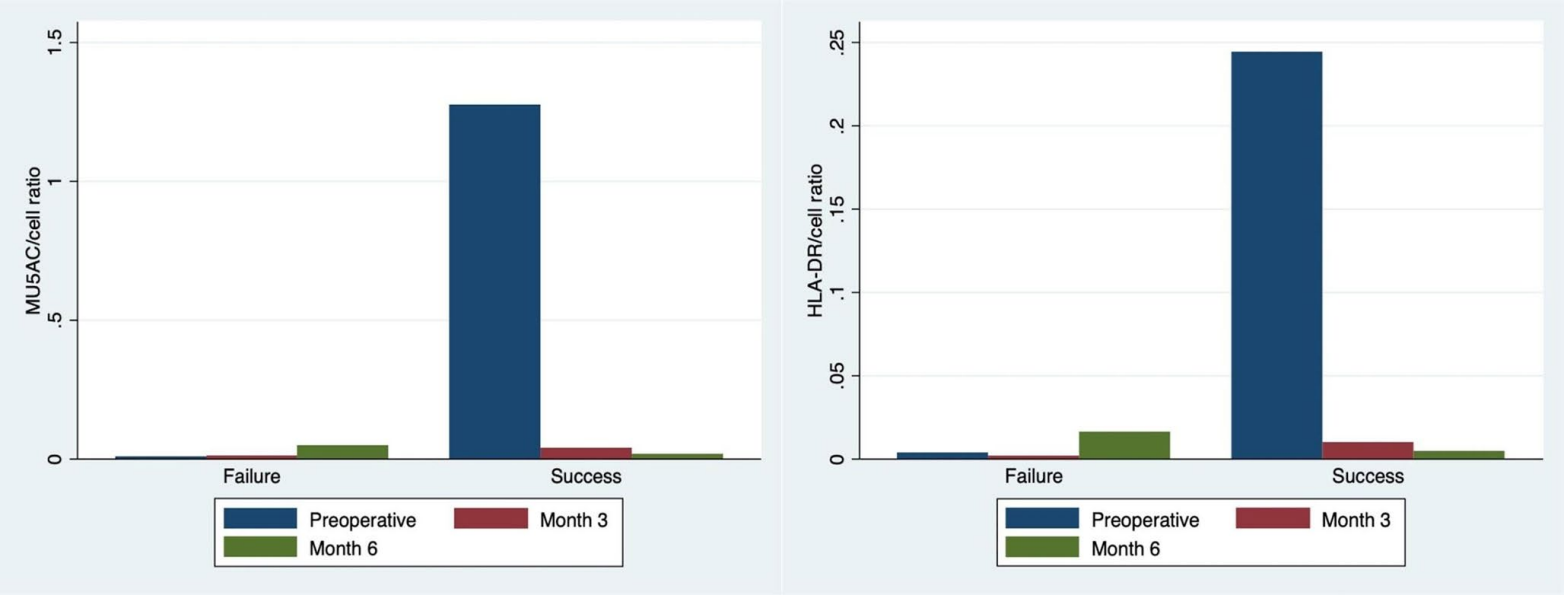
MU5AC/cell ratio (left) and HLA-DR/cell ratio (right) for both failed and successful groups

The ICA findings are summarized in Fig. 2 and SM 3. Overall, the conjunctiva showed a postoperative decline in the MUC5AC/cell ratio. Baseline MU5AC expression was higher in the success group. At 6 months, MUC5AC expression was significantly lower in the success group compared to the failure group (*p* = 0.02), with a borderline significant reduction in the MUC5AC/cell ratio (*p* = 0.06).

HLA-DR expression also declined over time, with the HLA-DR-cell area ratio significantly lower in the success group (*p* = 0.03). Interestingly, the failure group exhibited consistently low HLA-DR expression throughout the study period.

We analyzed the correlation between each postoperative parameter and the final measured IOP. Among all parameters, only the EMA on t IVCM, and the BECSA and BET on AS-OCT, showed significant correlations with final IOP. Notably, BECSA and BET demonstrated a moderate negative correlation with IOP (ρ_s_ = −0.51, *p* = 0.03 and ρ_s_ = −0.56, *p* = 0.02, respectively). Unexpectedly, EMA exhibited a positive correlation, indicating that a larger area of epithelial microcysts was associated with higher IOP. Scatterplots of each measurement are presented in SM4.

### Effect of bleb needling

Bleb needling was performed in 9 cases (see “Complications and postoperative interventions” section). The proportion of patients requiring needling was comparable between the success and failure groups. ICA did not show significant changes over time in the needling group, nor when compared to the non-needling group. However, postoperative imaging revealed differences in bleb characteristics following needling. AS-OCT imaging revealed differences between groups but only in the total area of the bleb subepithelial cysts (BSC); specifically, both the number and total area of BSCs were significantly lower in eyes that underwent needling. In contrast, IVCM appeared more sensitive to changes associated with needling. Compared to untreated “naïve” cases, eyes that received 5-FU needling demonstrated a non-significant reduction in SMR. GCD remained stable over time and did not differ significantly from the non-needling group. However, DCD, EMD, and area were significantly higher in the needling group and remained elevated through the six-month follow-up (see SM 5 to 7).

### Effect of pre- and intraoperative determining factors

In a subgroup analysis, we assessed the potential impact of cataract surgery on bleb parameters. Overall, no significant differences were observed in preoperative characteristics or during postoperative follow-up (see SM 8 to 10). AS-OCT measurements suggested epithelial changes associated with improved function in the combined surgery group, including an increase in epithelial and subepithelial microcysts, compared to stand-alone procedures. However, these findings were not corroborated by IVCM imaging.

To eliminate the potential confounding effect of previously reported preoperative factors (e.g., Diabetes Mellitus type 2 - DM2- and pseudoexfoliative glaucoma -PXG-, 8 cases were excluded (four with PEXG, four with DM2, and one with both conditions). A subsequent comparison between success and failure groups was then repeated. In this refined analysis, AS-OCT more consistently reflected the expected differences between successful and failed outcomes, whereas IVCM and ICA did not show corresponding changes. Nonetheless, most differences remained statistically nonsignificant, except for BESCD at the 6-month follow-up (SM 11 to 14).

## Discussion

The present study prospectively evaluated the conjunctival and morphological features of the XGS from baseline to 6-month follow-up, using four different approaches: slit lamp grading, AS-OCT, IVCM, and ICA. Our findings suggest that some postoperative changes already described in trabeculectomy – the gold standard glaucoma surgery- are also observed in this *ab interno* mitomycin-C augmented surgery. However, the resulting blebs were more homogeneous and flatter. Specifically, functioning XGS blebs tended to be less elevated and exhibited multiple cyst-like structures in the superficial layers, a higher baseline goblet cell density, and greater mucin content in the conjunctiva compared to unsuccessful surgeries.

Overall, the IOP and antiglaucoma medications were significantly reduced 6 months postoperatively, with a mean baseline IOP of 19.7 and 2.6 medications, compared to a mean final IOP of 16.3 and 0.6 medications. These results align with the few other prospective studies available, despite differences in sample size, surgical indications for XGS, and techniques (stand-alone vs. combined procedures). A comparison of our IOP and medication-lowering results to those obtained in other available prospective studies in the literature can be found in SM15 [4, 10, 14, 18–21]. Notably, our results are consistent with those of Arnould et al., [21] including the large standard deviation.

Our study also compared conjunctival changes and bleb morphology across the three imaging modalities. AS-OCT, a widely available imaging technique for qualitative and quantitative bleb analysis, has been used in only two prospective XGS studies [4, 18, 22]. Using Lenzhofer et al.’s classification [4], we identified that uniform blebs (type 1) were associated with surgical failure, while “subconjunctival separation” blebs (type 2) predominated in successful surgeries. Like their results, we found that the bleb morphology can change over the first six months, with an increased proportion of types 3 and 4 in the success group and of type 1 in the failure one.

AS-OCT has been associated with aqueous humor outflow through the conjunctiva and can be quantitatively measured. Sacchi and colleagues [12] introduced four new parameters (BECSD, BECSA, BSCSD, and BSCSA) to objectively characterize the filtration process in successful XGS and trabeculectomies. In our study, successful cases showed significantly larger epithelial and subepithelial cyst areas compared to failure cases, supporting the hypothesis that conjunctival aqueous humor diffusion serves as a biomarker for successful XGS filtering blebs [23]. 

IVCM and ICA have further elucidated ultrastructural conjunctival changes in XGS filtering blebs, including goblet and dendritic cell measurements. Previous studies have shown that IVCM and ICA can predict outcomes of glaucoma filtration surgery at baseline, differentiating success from failure, and complete versus qualified success in trabeculectomy [13, 17]. It has been hypothesized that superficial microcysts in filtering blebs represent goblet cells filled with aqueous humor instead of mucin [8]. In line with the works by Fea and Sacchi [12, 14], our IVCM analysis found that successful XGS surgeries were associated with a higher number of goblet cells and microcysts at baseline. However, in our study, only the epithelial microcyst area was statistically significant. Unlike Sacchi et al., [12] who observed a significant increase in microcyst number and area at 6 months, we found fewer microcysts but a larger filtering area, similar to Fea et al.’s measurements [14]. No significant differences in dendritic cell density were observed at baseline or during follow-up.

To further characterize cellular features, ICA was used to analyze mucin (goblet cells) and HLA-DR (dendritic cells) expression. We observed significantly higher mucin levels in the success group at 6 months (*p* < 0.05) but no statistically significant differences in HLA-DR expression during follow-up. Postoperatively, there was a reduction in goblet cell density and mucin expression, coupled with an increase in conjunctival microcysts, suggesting that aqueous humor diffusion through the conjunctiva is a key mechanism in XGS bleb functionality. However, baseline goblet cell density, mucin levels, dendritic cell density, and HLA-DR expression were not predictive of functioning versus non-functioning blebs. Factors such as preoperative topical glaucoma treatments (especially those with preservatives) or pre-existing dry eye disease, both of which can suppress GCD and mucin expression and elevate DCD levels in the ocular surface [24–27] may have influenced these outcomes.

AS-OCT, IVCM, and ICA detected changes in the bleb morphology in eyes requiring bleb needling with 5-FU. However, due to the lack of long-term comparative studies, needling decisions were not based on imaging findings. IVCM showed the most notable differences, including fewer dendritic cells, a wider epithelial microcyst area, and lower (non-significant) stromal reflectivity.

To date, only a limited number of studies have investigated conjunctival characteristics following bleb-filtering procedures, with even fewer focusing specifically on bleb-forming devices. While existing research has linked certain pre- and postoperative traits to surgical outcomes, the absence of standardized measured methods, cut-offs, and clinical trials guiding postoperative care has limited their clinical applicability. Additionally, studies such as that by Sacchi et al. [12] included patients with heterogeneous baseline characteristics, and both combined and standalone procedures. Certain baseline systemic and ocular factors, including DM2 and pseudoexfoliative glaucoma, have been associated with an increased likelihood of both clinical failure [28] and unfavourable bleb features [29, 30]. While these factors may serve as risk indicators for the surgeon and patient, it is ultimately the postoperative bleb appearance and the clinical management that should guide postoperative interventions. Therefore, identifying and characterizing bleb features postoperatively is essential for ensuring surgical success, regardless of preoperative risk factors. Similarly, combined phacoemulsification and filtering procedures are commonly performed, especially in elderly patients, due to the frequent coexistence of cataracts and glaucoma. With regard to XGS, existing literature has shown conflicting results -some studies have found differences between stand-alone and combined approaches [21] while others have not [31]. It has been suggested that phacoemulsification may increase proinflammatory mediators, potentially reducing bleb efficacy. However, only one study has evaluated this effect on bleb morphology, reporting similar bleb features between trabeculectomy and phaco-Express procedures, but fewer functional blebs in phaco-trabeculectomy, possibly implicating the proinflammatory role of iridectomy in addition to the cataract surgery itself [32]. Although our study population may be unbalanced, with fewer cases of DM2, PEXGs, and stand-alone procedures, the absence of significant differences between groups reinforces the notion that postoperative bleb characteristics are more critical to outcomes than baseline clinical factors.

This study has several limitations. While the use of three imaging modalities provides a more comprehensive understanding of XGS bleb function, applying all three in routine practice is neither feasible nor cost-effective. Additionally, each method has its limitations. IVCM and ICA can evaluate pre- and postoperative conjunctival changes and superficial bleb layers but cannot assess bleb morphology. Conversely, AS-OCT allows bleb morphology assessment but cannot capture preoperative features. Moreover, AS-OCT typically assesses a single vertical cross-section of the bleb, which may misrepresent overall bleb structure. Volumetric scans or bleb cube analyses, capturing intra- and superficial microcysts, may provide a more comprehensive evaluation of the entire bleb. The absence of standardized bleb parameters due to a lack of in-built analysis parameters or normative databases for these measurements. The reliance on semi-manual measurements, even with image analysis software, may contribute to variability between studies. Another potential source of bias could be the order of execution of the tests. There is no available evidence to determine which order of execution would have been the best. The most invasive procedure, the ICA, was performed last to avoid any histology factors on the conjunctiva, over other less invasive tests. However, had it been in the inverted order, the impact of ICA on other less invasive procedures would have been negligible, since the epithelial changes that could theoretically influence are described as mild and transient [33]. Other factors such as variations in surgical technique (e.g., MMC concentrations [4, 34], XGS positioning [35], and conjunctival approaches [36]). Finally, this study was conducted in the post-COVID era, which limited patient participation due to the need for lengthy pre- and postoperative visits.

Furthermore, during follow-up, AS-OCT alone may be sufficient to detect early morphological changes in these high-risk cases and help identify blebs at risk of failure. Ultimately, incorporating preoperative surface evaluation and postoperative imaging—particularly if supported by automated, standardized analysis tools—could support a more tailored surgical and medical management strategy to improve outcomes in XGS surgery.

In conclusion, clinically successful XGS blebs may exhibit higher baseline goblet cell density and mucin expression in the IVCM and ICA, respectively, as well as epithelial and subepithelial structural changes as detected by AS-OCT. Among the three imaging techniques used, AS-OCT was the most clinically applicable and showed the strongest correlation with the postoperative IOP.

While the combined use of AS-OCT, IVCM, and ICA is not practical for routine follow-up in all patients, this comprehensive approach may prove valuable in selected high-risk individuals (such as those with diabetes, pseudoexfoliation syndrome, African descent, or extensive prior topical treatment). Patients with preoperative features like reduced goblet cell density or low mucin expression may benefit from enhanced surface preparation, a more aggressive antifibrotic regimen, intensified postoperative steroid therapy, or even consideration of an open conjunctival surgical approach. In the postoperative period, AS-OCT alone may suffice to monitor bleb morphology and detect early signs of failure. The development of automated, built-in analysis tools for AS-OCT, replacing current semi-manual and non-standardized methods, could further enhance risk stratification and follow-up, regardless of baseline clinical features.

## Supplementary Information

Below is the link to the electronic supplementary material.

Supplementary Material 1

Supplementary Material 2

Supplementary Material 3

Supplementary Material 4

Supplementary Material 5

Supplementary Material 6

Supplementary Material 7

Supplementary Material 8

Supplementary Material 9

Supplementary Material 10

Supplementary Material 11

Supplementary Material 12

Supplementary Material 13

Supplementary Material 14

Supplementary Material 15

## Data Availability

Data are available upon reasonable request. No personal information that could reveal the identity of any of the participants will be transferred.

## References

[1] Abegao Pinto L, Sunaric Mégevand G, Stalmans I et al (2023) European glaucoma Society - A guide on surgical innovation for glaucoma. Br J Ophthalmol 107:1–114. 10.1136/bjophthalmol-2023-egsguidelines 38128960 10.1136/bjophthalmol-2023-egsguidelines

[2] Bicket AK, Le JT, Azuara-Blanco A et al (2021) Minimally invasive glaucoma surgical techniques for Open-Angle glaucoma: an overview of Cochrane systematic reviews and network Meta-analysis. JAMA Ophthalmol 139:983–989. 10.1001/jamaophthalmol.2021.2351 34264292 10.1001/jamaophthalmol.2021.2351 PMC8283665

[3] Park J, Rittiphairoj T, Wang X et al (2023) Device-modified trabeculectomy for glaucoma. Cochrane Database Syst Rev 3:CD010472. 10.1002/14651858.CD010472.pub3 36912740 10.1002/14651858.CD010472.pub3 PMC10010250

[4] Lenzhofer M, Strohmaier C, Hohensinn M et al (2019) Longitudinal bleb morphology in anterior segment OCT after minimally invasive transscleral Ab interno glaucoma gel microstent implantation. Acta Ophthalmol 97:e231–e237. 10.1111/aos.13902 30160048 10.1111/aos.13902 PMC6586011

[5] Picht G, Grehn F (1998) Classification of filtering blebs in trabeculectomy: biomicroscopy and functionality. Curr Opin Ophthalmol 9:2–8. 10.1097/00055735-199804000-00002 10180508 10.1097/00055735-199804000-00002

[6] Hoffmann EM, Herzog D, Wasielica-Poslednik J et al (2020) Bleb grading by photographs versus bleb grading by slit-lamp examination. Acta Ophthalmol 98:e607–e610. 10.1111/aos.14335 31889404 10.1111/aos.14335

[7] Singh M, Chew PTK, Friedman DS et al (2007) Imaging of trabeculectomy blebs using anterior segment optical coherence tomography. Ophthalmology 114:47–53. 10.1016/j.ophtha.2006.05.078 17070581 10.1016/j.ophtha.2006.05.078

[8] Amar N, Labbé A, Hamard P et al (2008) Filtering blebs and aqueous pathway an immunocytological and in vivo confocal microscopy study. Ophthalmology 115:1154–1161e4. 10.1016/j.ophtha.2007.10.024 18096232 10.1016/j.ophtha.2007.10.024

[9] Mills RP, Budenz DL, Lee PP et al (2006) Categorizing the stage of glaucoma from pre-diagnosis to end-stage disease. Am J Ophthalmol 141:24–30. 10.1016/j.ajo.2005.07.044 16386972 10.1016/j.ajo.2005.07.044

[10] Pérez-Torregrosa VT, Olate-Pérez Á, Cerdà-Ibáñez M et al (2016) Combined phacoemulsification and XEN45 surgery from a Temporal approach and 2 incisions. Arch Soc Esp Oftalmol 91:415–421. 10.1016/j.oftal.2016.02.006 26995503 10.1016/j.oftal.2016.02.006

[11] Wells AP, Crowston JG, Marks J et al (2004) A pilot study of a system for grading of drainage blebs after glaucoma surgery. J Glaucoma 13:454–460. 10.1097/00061198-200412000-00005 15534469 10.1097/00061198-200412000-00005

[12] Sacchi M, Agnifili L, Brescia L et al (2020) Structural imaging of conjunctival filtering blebs in XEN gel implantation and trabeculectomy: a confocal and anterior segment optical coherence tomography study. Graefes Arch Clin Exp Ophthalmol 258:1763–1770. 10.1007/s00417-020-04671-2 32415535 10.1007/s00417-020-04671-2

[13] Mastropasqua R, Fasanella V, Brescia L et al (2017) In vivo confocal imaging of the conjunctiva as a predictive tool for the glaucoma filtration surgery outcome. Invest Ophthalmol Vis Sci 58:BIO114–BIO120. 10.1167/iovs.17-21795 28586797 10.1167/iovs.17-21795

[14] Fea AM, Spinetta R, Cannizzo PML et al (2017) Evaluation of bleb morphology and reduction in IOP and glaucoma medication following implantation of a novel gel stent. J Ophthalmol 2017:9364910. 10.1155/2017/9364910 28751986 10.1155/2017/9364910 PMC5511657

[15] Kok PHB, van den Berg TJTP, van Dijk HW et al (2013) The relationship between the optical density of cataract and its influence on retinal nerve fibre layer thickness measured with spectral domain optical coherence tomography. Acta Ophthalmol 91:418–424. 10.1111/j.1755-3768.2012.02514.x 23106951 10.1111/j.1755-3768.2012.02514.x

[16] Efron N, Al-Dossari M, Pritchard N (2009) In vivo confocal microscopy of the bulbar conjunctiva. Clin Exp Ophthalmol 37:335–344. 10.1111/j.1442-9071.2009.02065.x 19594558 10.1111/j.1442-9071.2009.02065.x

[17] Agnifili L, Fasanella V, Mastropasqua R et al (2016) In vivo goblet cell density as a potential indicator of glaucoma filtration surgery outcome. Invest Ophthalmol Vis Sci 57:2928–2935. 10.1167/iovs.16-19257 27249666 10.1167/iovs.16-19257

[18] Galal A, Bilgic A, Eltanamly R, Osman A (2017) XEN glaucoma implant with mitomycin C 1-Year Follow-Up: result and complications. J Ophthalmol 2017:5457246. 10.1155/2017/5457246 28348884 10.1155/2017/5457246 PMC5350531

[19] Reitsamer H, Sng C, Vera V et al (2019) Two-year results of a multicenter study of the Ab interno gelatin implant in medically uncontrolled primary open-angle glaucoma. Graefes Arch Clin Exp Ophthalmol 257:983–996. 10.1007/s00417-019-04251-z 30758653 10.1007/s00417-019-04251-z

[20] Oddone F, Roberti G, Giammaria S et al (2024) Effectiveness and safety of XEN45 implant over 12 months of follow-up: data from the XEN-Glaucoma treatment registry. Eye (Lond) 38:103–111. 10.1038/s41433-023-02642-5 37414935 10.1038/s41433-023-02642-5 PMC10764778

[21] Arnould L, Balsat E, Hashimoto Y et al (2024) Two-year outcomes of Xen 45 gel stent implantation in patients with open-angle glaucoma: real-world data from the fight glaucoma blindness registry. Br J Ophthalmol 108:1672–1678. 10.1136/bjo-2023-325077 38789132 10.1136/bjo-2023-325077 PMC11671995

[22] Kudsieh B, Fernández-Vigo JI, Canut Jordana MI et al (2022) Updates on the utility of anterior segment optical coherence tomography in the assessment of filtration blebs after glaucoma surgery. Acta Ophthalmol 100:e29–e37. 10.1111/aos.14881 33942540 10.1111/aos.14881

[23] Teus MA, Paz Moreno-Arrones J, Castaño B et al (2019) Optical coherence tomography analysis of filtering blebs after long-term, functioning trabeculectomy and XEN^®^ stent implant. Graefes Arch Clin Exp Ophthalmol 257:1005–1011. 10.1007/s00417-019-04272-8 30783784 10.1007/s00417-019-04272-8

[24] Mastropasqua R, Agnifili L, Fasanella V et al (2016) In vivo distribution of corneal epithelial dendritic cells in patients with glaucoma. Invest Ophthalmol Vis Sci 57:5996–6002. 10.1167/iovs.16-20333 27820631 10.1167/iovs.16-20333

[25] Bron AJ, de Paiva CS, Chauhan SK et al (2017) TFOS DEWS II pathophysiology report. Ocul Surf 15:438–510. 10.1016/j.jtos.2017.05.011 28736340 10.1016/j.jtos.2017.05.011

[26] Duan H, Yang T, Zhou Y et al (2023) Comparison of mucin levels at the ocular surface of visual display terminal users with and without dry eye disease. BMC Ophthalmol 23:189. 10.1186/s12886-023-02931-3 37106448 10.1186/s12886-023-02931-3 PMC10139827

[27] Kolko M, Gazzard G, Baudouin C et al (2023) Impact of glaucoma medications on the ocular surface and how ocular surface disease can influence glaucoma treatment. Ocul Surf 29:456–468. 10.1016/j.jtos.2023.05.012 37302545 10.1016/j.jtos.2023.05.012

[28] Schlenker MB, Gulamhusein H, Conrad-Hengerer I et al (2017) Efficacy, safety, and risk factors for failure of standalone Ab interno gelatin microstent implantation versus standalone trabeculectomy. Ophthalmology 124:1579–1588. 10.1016/j.ophtha.2017.05.004 28601250 10.1016/j.ophtha.2017.05.004

[29] Erdoğan H, Arici DS, Toker MI et al (2006) Conjunctival impression cytology in pseudoexfoliative glaucoma and pseudoexfoliation syndrome. Clin Exp Ophthalmol 34:108–113. 10.1111/j.1442-9071.2006.01168.x 16626422 10.1111/j.1442-9071.2006.01168.x

[30] Sahu J, Vallinayagam M, Srikanth K, Srini S (2024) Conjunctival impression cytology and tear film changes in patients with type 2 diabetes mellitus and its correlation with severity of diabetic retinopathy. Saudi J Ophthalmol. 10.4103/sjopt.sjopt_214_23 40182977 10.4103/sjopt.sjopt_214_23 PMC11964339

[31] Reitsamer H, Vera V, Ruben S et al (2022) Three-year effectiveness and safety of the XEN gel stent as a solo procedure or in combination with phacoemulsification in open-angle glaucoma: a multicentre study. Acta Ophthalmol 100:e233–e245. 10.1111/aos.14886 33973370 10.1111/aos.14886 PMC9290976

[32] Zhang Y, Lai C, Zhao S et al (2024) Comparison of bleb morphologies between phacoemulsification combined with Ex-PRESS mini shunt implantation, phacotrabeculectomy and trabeculectomy alone: a two-year retrospective in vivo confocal microscopy study. BMC Ophthalmol 24:108. 10.1186/s12886-024-03364-2 38448910 10.1186/s12886-024-03364-2 PMC10916144

[33] Calonge M, Diebold Y, Sáez V et al (2004) Impression cytology of the ocular surface: A review. Exp Eye Res 78:457–472. 10.1016/j.exer.2003.09.009 15106925 10.1016/j.exer.2003.09.009

[34] Kim Y, Lim S-H, Rho S (2022) Bleb analysis using anterior segment optical coherence tomography and surgical predictors of XEN gel stent. Transl Vis Sci Technol 11:26. 10.1167/tvst.11.2.26 35171225 10.1167/tvst.11.2.26 PMC8857613

[35] Lenzhofer M, Strohmaier C, Sperl P et al (2019) Effect of the outer stent position on efficacy after minimally invasive transscleral glaucoma gel stent implantation. Acta Ophthalmol 97:e1105–e1111. 10.1111/aos.14167 31210015 10.1111/aos.14167 PMC6899703

[36] Hasan SM, Theilig T, Tarhan M et al (2023) [Bleb morphology using optical coherence tomography : after primary implantation of XEN gel stent and open conjunctival revision]. Die Ophthalmologie 120:529–537. 10.1007/s00347-022-01764-7 36445475 10.1007/s00347-022-01764-7

