# Supplementary material for: Longitudinal analysis of XEN45 gel stent bleb morphology using bleb grading scales, anterior segment-OCT, in vivo confocal microscopy, and impression cytology
Source: Graefes Arch Clin Exp Ophthalmol. 2025 Oct 3;264(1):207–18. doi: 10.1007/s00417-025-06952-0 (PMC12906558; doi:10.1007/s00417-025-06952-0)
Supplement: Supplementary file 2 — Supplementary Material 2 [file 417_2025_6952_MOESM2_ESM.docx]

|  | Baseline BCVA | M3 BCVA | M6 BCVA | Baseline ECD | M6 ECD | Baseline MD | Postop^Ω^ MD | Baseline pRNFL | Postop^Ω^ pRNFL |
| --- | --- | --- | --- | --- | --- | --- | --- | --- | --- |
| XGS | 0.69 (0.22) | 0.75 (0.21) | 0.81 (0.18)^a^ | 1968 (583) | 1621 (489)^b^ | -4.48 (4.01) | -5.14 (4.41) | 72.0 (10.6) | 78.00 (10.34)^a^ |

Supplementary Material 2. Visual acuity, endothelial cell count, and glaucoma parameters over the follow-up period. ECD (cells/mm^2^): Endothelial Cell Count; MD = mean deviation; pRNFL = peripapillary Retinal Nerve Fiber Layer (µm); XGS: XEN45 Gel Stent. ^Ω^ performed 3 to 6 months postoperatively. All values are expressed as mean (standard deviation); ^a^ p<0.05 vs baseline, paired t-test; ^b^ p<0.05 vs baseline, Wilcoxon rank sum test.
