# Supplementary material for: Longitudinal analysis of XEN45 gel stent bleb morphology using bleb grading scales, anterior segment-OCT, in vivo confocal microscopy, and impression cytology
Source: Graefes Arch Clin Exp Ophthalmol. 2025 Oct 3;264(1):207–18. doi: 10.1007/s00417-025-06952-0 (PMC12906558; doi:10.1007/s00417-025-06952-0)
Supplement: Supplementary file 3 — Supplementary Material 3 [file 417_2025_6952_MOESM3_ESM.docx]

|  | MUC5AC/cell | | | | HLA-DR/cell | | | |
| --- | --- | --- | --- | --- | --- | --- | --- | --- |
| Mean (SD) | Preop | M3 | M6 | p value** | Preop | M3 | M6 | p value** |
| Overall | 0.828 (3.334) | 0.024 (0.055) | 0.032 (0.032) | 0.36 | 0.159 (0.614) | 0.006 (0.012) | 0.010 (0.015) | 0.34 |
| Success | 1.276 (4.143) | 0.039 (0.080) | 0.017 (0.012) | 0.40 | 0.244 (0.762) | 0.010 (0.012) | 0.005 (0.005) | 0.38 |
| Failure | 0.008 (0.006) | 0.013 (0.021) | 0.048 (0.039) | 0.10 | 0.004 (0.003) | 0.002 (0.001) | 0.016 (0.020) | 0.32 |
| p value* | 0.11 | 1.00 | **0.02** | **--** | 0.16 | 0.58 | 0.53 | -- |

Supplementary material 3. Impression Cytology Analysis: mucin and HLADR levels were expressed as the area covered by MUC5AC staining and the area covered by HLADR staining divided by the area occupied by cells, respectively. * Mann-Whitney U-test, comparing success vs failure cases. **ANOVA for repeated measures
