# Supplementary figures and images for: Longitudinal analysis of XEN45 gel stent bleb morphology using bleb grading scales, anterior segment-OCT, in vivo confocal microscopy, and impression cytology
Source: Graefes Arch Clin Exp Ophthalmol. 2025 Oct 3;264(1):207–18. doi: 10.1007/s00417-025-06952-0 (PMC12906558; doi:10.1007/s00417-025-06952-0)

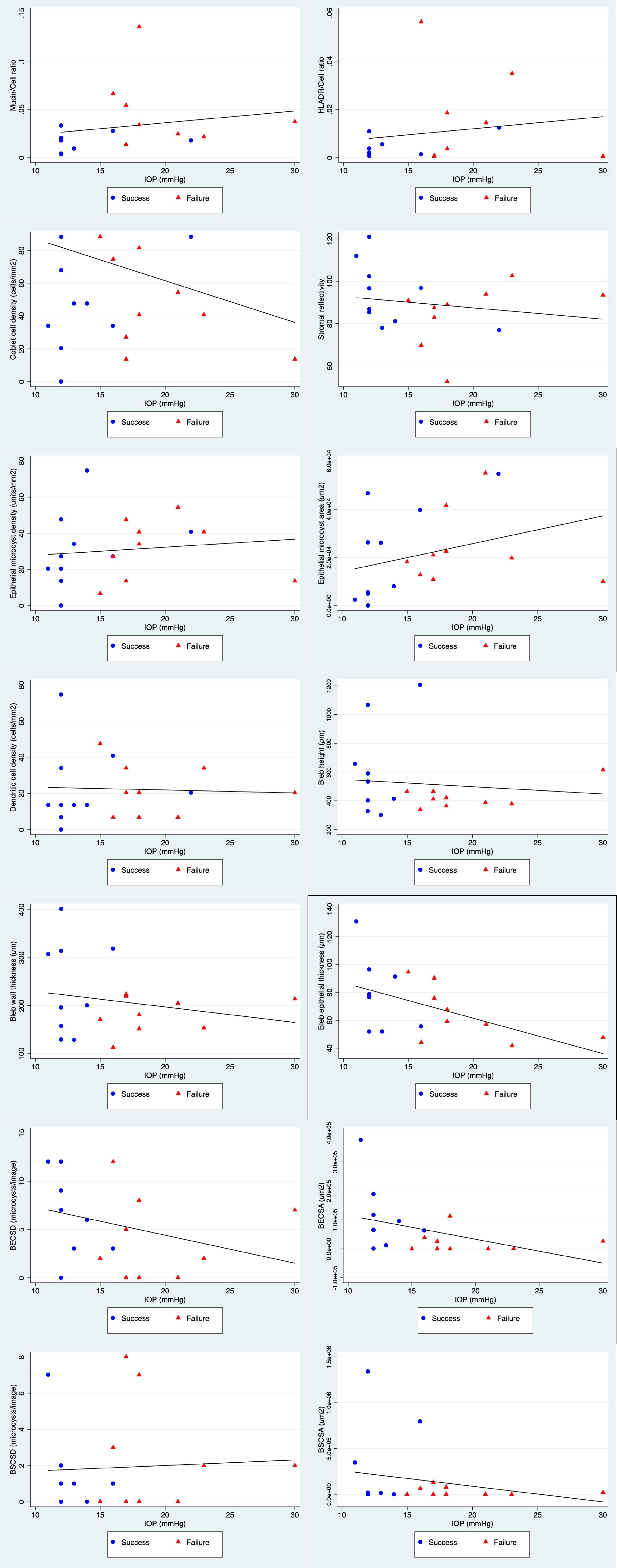

Supplement: Supplementary file 4 — Supplementary Material 4 [file 417_2025_6952_MOESM4_ESM.jpg]
