# Supplementary material for: Longitudinal analysis of XEN45 gel stent bleb morphology using bleb grading scales, anterior segment-OCT, in vivo confocal microscopy, and impression cytology
Source: Graefes Arch Clin Exp Ophthalmol. 2025 Oct 3;264(1):207–18. doi: 10.1007/s00417-025-06952-0 (PMC12906558; doi:10.1007/s00417-025-06952-0)
Supplement: Supplementary file 5 — Supplementary Material 5 [file 417_2025_6952_MOESM5_ESM.docx]

|  | MUC5AC/cell | | | | HLA-DR/cell | | | |
| --- | --- | --- | --- | --- | --- | --- | --- | --- |
| Mean (SD) | Preop | M3 | M6 | p value** | Preop | M3 | M6 | p value** |
| Overall | 0.828 (3.334) | 0.024 (0.055) | 0.032 (0.032) | 0.36 | 0.159 (0.614) | 0.006 (0.012) | 0.010 (0.015) | 0.34 |
| Needling | 1.982 (5.197) | 0.004 (0.002) | 0.026 (0.015) | 0.38 | 0.372 (0.958) | 0.003 (0.001) | 0.012 (0.012) | 0.37 |
| No needling | 0.022 (0.032) | 0.042 (0.072) | 0.037 (0.042) | 0.78 | 0.011 (0.012) | 0.001 (0.017) | 0.009 (0.018) | 0.26 |
| p value* | 0.27 | 0.47 | 0.79 | **--** | 0.19 | 0.27 | 0.43 | -- |

Supplementary material 3. Impression Cytology Analysis: mucin and HLADR levels were expressed as the area covered by MUC5AC staining and the area covered by HLADR staining divided by the area occupied by cells, respectively. * Mann-Whitney U-test, comparing needling vs no needling. **ANOVA for repeated measures
