# Supplementary material for: Longitudinal analysis of XEN45 gel stent bleb morphology using bleb grading scales, anterior segment-OCT, in vivo confocal microscopy, and impression cytology
Source: Graefes Arch Clin Exp Ophthalmol. 2025 Oct 3;264(1):207–18. doi: 10.1007/s00417-025-06952-0 (PMC12906558; doi:10.1007/s00417-025-06952-0)
Supplement: Supplementary file 6 — Supplementary Material 6 [file 417_2025_6952_MOESM6_ESM.docx]

|  | BECSD | | | BECSA | | | BSCSD | | | BSCSA | | |
| --- | --- | --- | --- | --- | --- | --- | --- | --- | --- | --- | --- | --- |
|  | M1 | M3 | M6 | M1 | M3 | M6 | M1 | M3 | M6 | M1 | M3 | M6 |
| Overall | 8.2 (5.5) | 5.5 (4.3) | 5.6 (4.5) | 41321.8 (36696.5) | 33115.9 (36125.6) | 65658.4 (93729.6) | 4.7 (4.4) | 3.9 (4.2) | 1.9 (2.7) | 176599.5 (277716.2) | 251195.8 (476213.2) | 155215.4 (354701.1) |
| Needling | 5.3 (4.8) | 4.8 (4.0) | 3.7 (4.2) | 27488.9 (31950.8) | 31798.1 (46820.9) | 50958.3 (68545.5) | 3.4 (4.8) | 2.2 (3.5) | 1.1 (2.3) | 78377.0 (121642.1) | 172666.2 (474643.2) | 97007.0 (263180.6) |
| No needling | 10.7 (4.9) | 6.2 (4.8) | 7.4 (4.3) | 52639.5 (37799.6) | 34433.7 (24023.9) | 80358.5 (116104.9) | 5.8 (4.0) | 5.6 (4.3) | 2.7 (2.9) | 264999.8 (350384.5) | 329725.4 (492690.2) | 213423.9 (436422.7) |
| p value* | 0.03 | 0.56 | 0.07 | 0.10 | 0.42 | 0.35 | 0.1 | 0.06 | 0.08 | **0.04** | **0.04** | 0.08 |

(cont.)

|  | BH | | | BT | | | BET | | |
| --- | --- | --- | --- | --- | --- | --- | --- | --- | --- |
|  | M1 | M3 | M6 | M1 | M3 | M6 | M1 | M3 | M6 |
| Overall | 562.7 (167.5) | 541.4 (210.8) | 519.1 (246.7) | 230.5 (86.3) | 212.5 (83.0) | 210.1 (78.1) | 78.8 (21.9) | 72.2 (17.2) | 71.5 (23.2) |
| Needling | 558.7 (187.8) | 584.9 (197.7) | 517.2 (268.7) | 201.8 (43.6) | 205.0 (90.8) | 209.9 (64.9) | 76.6 (27.9) | 72.1 (21.6) | 67.1 (18.4) |
| No needling | 566.4 (157.3) | 498.0 (226.0) | 520.9 (239.0) | 256.4 (107.9) | 220.0 (79.1) | 210.2 (93.6) | 80.9 (15.9) | 72.4 (12.6) | 76.0 (27.6) |
| p value* | 0.68 | 0.20 | 0.89 | 0.25 | 0.51 | 0.76 | 0.57 | 0.96 | 0.57 |

Table 2. Anterior Segment OCT (AS-OCT) bleb quantitative measurements over time.

BECSA, bleb-wall epithelium cyst-like structure area (µm^2^); BECSD, bleb-wall epithelium cyst-like structure density (microcysts/image); BSCSA, bleb-wall sub-epithelium cyst-like structure area(µm^2^); BSCSD, bleb-wall sub-epithelium cyst-like structure density (microcysts/image); BH, Bleb Height (µm); BT, Bleb Thickness (µm); BET, Bleb Epithelial Thickness (µm); * Mann-Whitney U-test (needling/no-needling comparison).
