# Supplementary material for: Longitudinal analysis of XEN45 gel stent bleb morphology using bleb grading scales, anterior segment-OCT, in vivo confocal microscopy, and impression cytology
Source: Graefes Arch Clin Exp Ophthalmol. 2025 Oct 3;264(1):207–18. doi: 10.1007/s00417-025-06952-0 (PMC12906558; doi:10.1007/s00417-025-06952-0)
Supplement: Supplementary file 7 — Supplementary Material 7 [file 417_2025_6952_MOESM7_ESM.docx]

|  | MUC5AC/cell | | | | HLA-DR/cell | | | |
| --- | --- | --- | --- | --- | --- | --- | --- | --- |
| Mean (SD) | Preop | M3 | M6 | p value** | Preop | M3 | M6 | p value** |
| Overall | 1.386 (4.350) | 0.003 (0.002) | 0.039 (0.038) | 0.37 | 0.259 (0.803) | 0.002 (0.001) | 0.005 (0.006) | 0.37 |
| Success | 1.731 (4.863) | 0.004 (0.002) | 0.37 (0.041) | 0.38 | 0.322 (0.898) | 0.002 (0.001) | 0.006 (0.007) | 0.38 |
| Failure | 0.005 (0.001) | 0.001 (0.003) | 0.054 (0.031) | 0.4 | 0.006 (0.001) | 0.004 (0.002) | 0.006 (0.005) | 0.85 |
| p value* | 0.19 | 0.14 | 0.25 | **--** | 0.60 | 0.14 | 0.12 | -- |

Supplementary material 3. Impression Cytology Analysis: mucin and HLADR levels were expressed as the area covered by MUC5AC staining and the area covered by HLADR staining divided by the area occupied by cells, respectively. * Mann-Whitney U-test, comparing needling vs no needling. **ANOVA for repeated measures
