# Supplementary material for: Longitudinal analysis of XEN45 gel stent bleb morphology using bleb grading scales, anterior segment-OCT, in vivo confocal microscopy, and impression cytology
Source: Graefes Arch Clin Exp Ophthalmol. 2025 Oct 3;264(1):207–18. doi: 10.1007/s00417-025-06952-0 (PMC12906558; doi:10.1007/s00417-025-06952-0)
Supplement: Supplementary file 8 — Supplementary Material 8 [file 417_2025_6952_MOESM8_ESM.docx]

|  | SMR | | | | GCD | | | | EMD | | | | |
| --- | --- | --- | --- | --- | --- | --- | --- | --- | --- | --- | --- | --- | --- |
| Mean (SD) | Preop | M3 | M6 | p value ** | Preop | M3 | M6 | p value ** | Preop | M3 | M6 | p value ** |  |
| Overall | 103.3 (18.9) | 85.6 (17.5) | 89.7 (6.4) | 0.07 | 55.4 (40.6) | 45.0 (28.3) | 54.3 (27.8) | 0.41 | 23.2 (11.4) | 31.4 (15.5) | 34.5 (21.5) | 0.19 |  |
| Success | 108.4 (19.6) | 88.0 (20.0) | 95.5 (14.7) | 0.24 | 64.9 (47.1) | 40.7 (29.4) | 50.1 (31.2) | 0.24 | 28.1 (10.7) | 39.6 (15.7) | 32.8 (25.5) | 0.56 |  |
| Failure | 96.2 (17.2) | 82.7 (15.7) | 82.7 (17.0) | 0.28 | 42.0 (28.5) | 50.2 (29.4) | 58.3 (26.1) | 0.14 | 16.3 (9.1) | 21.7 (8.8) | 36.6 (18.3) | 0.12 |  |
| p value* | 0.22 | 0.27 | 0.58 |  | 0.46 | 0.78 | 0.78 |  | 0.07 | 0.06 | 0.52 |  |  |

|  | EMA | | | | DCD | | | |
| --- | --- | --- | --- | --- | --- | --- | --- | --- |
| Mean (SD) | Preop | M3 | M6 | p value ** | Preop | M3 | M6 | p value ** |
| Overall | 6175.0 (4965.0) | 28475.0 (37213.0) | 21866.7 (19356.0) | 0.09 | 14.1 (10.2) | 25.7 (26.8) | 24.0 (22.8) | 0.32 |
| Success | 8628.6 (5288.9) | 28171.4 (40180.6) | 14914.3 (19538.6) | 0.26 | 17.4 (11.7) | 21.5 (20.8) | 24.9 (28.0) | 0.72 |
| Failure | 2740.1 (740.3) | 28900.0 (37230.4) | 31600.8 (15958.1) | 0.23^**^ | 9.5 (6.1) | 29.8 (34.8) | 23.1 (17.7) | 0.26 |
| p value* | **0.01** | 0.94 | 0.09 |  | 0.18 | 0.93 | 0.85 |  |

Table 3. In Vivo Confocal Microscopy (IVCM) Study Findings during the follow-up. 3G. * Mann-Whitney U-test, comparing success vs failure. DCD: dendritic cell density (cells/mm^2^); EMD: epithelial microcyst density (microcysts/mm^2^); EMA: epithelial microcyst area (µm^2^); GCD: goblet cell density (cells/mm^2^); M3: month3; M6: month 6; SMR: stromal meshwork reflectivity (arbitrary scale). **ANOVA for repeated measures. ^#^ EMD was significantly higher when comparing the preoperative and the 3-month visit. ^##^ EMA was significantly higher in both postoperative visits
