# Supplementary material for: Longitudinal analysis of XEN45 gel stent bleb morphology using bleb grading scales, anterior segment-OCT, in vivo confocal microscopy, and impression cytology
Source: Graefes Arch Clin Exp Ophthalmol. 2025 Oct 3;264(1):207–18. doi: 10.1007/s00417-025-06952-0 (PMC12906558; doi:10.1007/s00417-025-06952-0)
Supplement: Supplementary file 9 — Supplementary Material 9 [file 417_2025_6952_MOESM9_ESM.docx]

|  | BECSD | | | BECSA | | | BSCSD | | | BSCSA | | |
| --- | --- | --- | --- | --- | --- | --- | --- | --- | --- | --- | --- | --- |
|  | M1 | M3 | M6 | M1 | M3 | M6 | M1 | M3 | M6 | M1 | M3 | M6 |
| Overall | 8.2 (5.5) | 5.5 (4.3) | 5.6 (4.5) | 41321.8 (36696.5) | 33115.9 (36125.6) | 65658.4 (93729.6) | 4.7 (4.4) | 3.9 (4.2) | 1.9 (2.7) | 176599.5 (277716.2) | 251195.8 (476213.2) | 155215.4 (354701.1) |
| Combined | 8.1 (6.2) | 4.8 (3.9) | 6.1 (4.8) | 35413.8 (33914.4) | 37818.1 (41167.3) | 75714.93 (102584.3) | 5.2 (4.8) | 4.8 (4.5) | 2.3 (2.9) | 204913.4 (315094.6) | 285439.5 (529373.8) | 198185.2 (3944404.7) |
| Standalone | 8.2 (3.1) | 7.2 (5.4) | 3.8 (3.3) | 59045.61 (42980.26) | 20890.1 (14267.7) | 30460.5 (45251.7) | 3.2 (3.1) | 1.4 (1.9) | 0.5 (1.0) | 97320.8 (117179.6) | 162162.3 (330728.1) | 4821.2 (9642.5) |
| p value* | 0.89 | 0.43 | 0.36 | 0.23 | 0.55 | 0.33 | 0.43 | 0.12 | 0.20 | 0.52 | 0.32 | 0.16 |

(cont.)

|  | BH | | | BT | | | BET | | |
| --- | --- | --- | --- | --- | --- | --- | --- | --- | --- |
|  | M1 | M3 | M6 | M1 | M3 | M6 | M1 | M3 | M6 |
| Overall | 562.7 (167.5) | 541.4 (210.8) | 519.1 (246.7) | 230.5 (86.3) | 212.5 (83.0) | 210.1 (78.1) | 78.8 (21.9) | 72.2 (17.2) | 71.5 (23.2) |
| Combined | 540.8 (188.6) | 507.7 (226.2) | 527.4 (278.4) | 237.0 (97.8) | 192.6 (72.4) | 212.6 (88.6) | 76.2 (21.1) | 72.0 (16.5) | 69.9 (24.2) |
| Standalone | 624.3 (65.4) | 629.0 (148.4) | 489.9 (86.5) | 212.3 (43.3) | 264.2 (94.2) | 201.1 (21.7) | 86.0 (24.8) | 72.8 (20.9) | 77.4 (21.4) |
| p value* | 0.12 | 0.13 | 0.34 | 0.71 | 0.10 | 0.67 | 0.46 | 0.88 | 0.52 |

Table 2. Anterior Segment OCT (AS-OCT) bleb quantitative measurements over time.

BECSA, bleb-wall epithelium cyst-like structure area (µm^2^); BECSD, bleb-wall epithelium cyst-like structure density (microcysts/image); BSCSA, bleb-wall sub-epithelium cyst-like structure area(µm^2^); BSCSD, bleb-wall sub-epithelium cyst-like structure density (microcysts/image); BH, Bleb Height (µm); BT, Bleb Thickness (µm); BET, Bleb Epithelial Thickness (µm); * Mann-Whitney U-test (combined vs standalone procedure).
