# Supplementary material for: Longitudinal analysis of XEN45 gel stent bleb morphology using bleb grading scales, anterior segment-OCT, in vivo confocal microscopy, and impression cytology
Source: Graefes Arch Clin Exp Ophthalmol. 2025 Oct 3;264(1):207–18. doi: 10.1007/s00417-025-06952-0 (PMC12906558; doi:10.1007/s00417-025-06952-0)
Supplement: Supplementary file 10 — Supplementary Material 10 [file 417_2025_6952_MOESM10_ESM.docx]

|  | SMR | | | | GCD | | | | EMD | | | | |
| --- | --- | --- | --- | --- | --- | --- | --- | --- | --- | --- | --- | --- | --- |
| Mean (SD) | Preop | M3 | M6 | p value ** | Preop | M3 | M6 | p value ** | Preop | M3 | M6 | p value ** |  |
| Overall | 96.0 (27.9) | 89.8 (17.9) | 89.4 (15.2) | 0.43 | 55.2 (45.6) | 53.8 (43.4) | 48.9 (27.7) | 0.86 | 21.6 (12.3) | 36.2 (19.9) | 30.7 (18.3) | 0.03^#^ |  |
| Needling | 101.3 (16.4) | 85.1 (19.5) | 87.4 (14.6) | 0.14 | 59.5 (42.3) | 49.0 (28.7) | 49.0 (24.2) | 0.45 | 19.6 (11.0) | 39.9 (19.3) | 38.4 (20.9) | **0.04** |  |
| No needling | 92.3 (36.0) | 94.5 (16.0) | 91.2 (16.2) | 0.34 | 40.1 (28.5) | 58.7 (55.9) | 48.8 (31.8) | 0.60 | 21.6 (12.8) | 32.4 (20.8) | 23.7 (12.9) | 0.40 |  |
| p value* | 0.57 | 0.40 | 0.87 |  | 0.32 | 0.82 | 0.93 |  | 0.62 | 0.37 | 0.09 |  |  |

|  | EMA | | | | DCD | | | |
| --- | --- | --- | --- | --- | --- | --- | --- | --- |
| Mean (SD) | Preop | M3 | M6 | p value ** | Preop | M3 | M6 | p value ** |
| Overall | 7281.0 (6087.7) | 25976.2 (30819.9) | 20228.6 (17858.1) | 0.01^##^ | 15.3 (11.0) | 24.9 (22.2) | 22.5 (18.1) | 0.26 |
| Needling | 6322.2 (5063.3) | 40888.9 (39881.0) | 30588.9 (15585.5) | **0.03^**^** | 14.3 (12.3) | 36.2 (25.8) | 32.4 (21.7) | **0.04** |
| No needling | 8218.2 (7178.7) | 16136.4 (15912.3) | 13590.9 (16047.2) | 0.11^**^ | 16.3 (18.1) | 13.6 (9.6) | 13.6 (7.2) | 0.42 |
| p value* | 0.49 | 0.27 | **0.04** |  | 0.91 | 0.06 | **0.05** |  |

Table 3. In Vivo Confocal Microscopy (IVCM) Study Findings during the follow-up. 3G. * Mann-Whitney U-test, comparing needling vs no-needling cases. DCD: dendritic cell density (cells/mm^2^); EMD: epithelial microcyst density (microcysts/mm^2^); EMA: epithelial microcyst area (µm^2^); GCD: goblet cell density (cells/mm^2^); M3: month3; M6: month 6; SMR: stromal meshwork reflectivity (arbitrary scale). **ANOVA for repeated measures. ^#^ EMD was significantly higher when comparing the preoperative and the 3-month visit. ^##^ EMA was significantly higher in both postoperative visits
