# Supplementary material for: Longitudinal analysis of XEN45 gel stent bleb morphology using bleb grading scales, anterior segment-OCT, in vivo confocal microscopy, and impression cytology
Source: Graefes Arch Clin Exp Ophthalmol. 2025 Oct 3;264(1):207–18. doi: 10.1007/s00417-025-06952-0 (PMC12906558; doi:10.1007/s00417-025-06952-0)
Supplement: Supplementary file 11 — Supplementary Material 11 [file 417_2025_6952_MOESM11_ESM.docx]

|  | MUC5AC/cell | | | | HLA-DR/cell | | | |
| --- | --- | --- | --- | --- | --- | --- | --- | --- |
| Mean (SD) | Preop | M3 | M6 | p value** | Preop | M3 | M6 | p value** |
| Overall | 0.828 (3.334) | 0.024 (0.055) | 0.032 (0.032) | 0.36 | 0.159 (0.614) | 0.006 (0.012) | 0.010 (0.015) | 0.34 |
| Combined | 1.164 (3.969) | 0.004 (0.003) | 0.031 (0.035) | 0.37 | 0.220 (0.732) | 0.002 (0.001) | 0.012 (0.017) | 0.36 |
| Standalone | 0.028 (0.047) | 0.080 (0.093) | 0.036 (0.018) | 0.53 | 0.015 (0.012) | 0.015 (0.023) | 0.004 (0.007) | 0.22 |
| p value* | 0.40 | 0.31 | 0.38 | **--** | 0.63 | 0.22 | 0.16 | -- |

Supplementary material 3. Impression Cytology Analysis: mucin and HLADR levels were expressed as the area covered by MUC5AC staining and the area covered by HLADR staining divided by the area occupied by cells, respectively. * Mann-Whitney U-test, comparing combined vs standalone procedures. **ANOVA for repeated measures
