# Supplementary material for: Longitudinal analysis of XEN45 gel stent bleb morphology using bleb grading scales, anterior segment-OCT, in vivo confocal microscopy, and impression cytology
Source: Graefes Arch Clin Exp Ophthalmol. 2025 Oct 3;264(1):207–18. doi: 10.1007/s00417-025-06952-0 (PMC12906558; doi:10.1007/s00417-025-06952-0)
Supplement: Supplementary file 13 — Supplementary Material 13 [file 417_2025_6952_MOESM13_ESM.docx]

|  | SMR | | | | GCD | | | | EMD | | | | |
| --- | --- | --- | --- | --- | --- | --- | --- | --- | --- | --- | --- | --- | --- |
| Mean (SD) | Preop | M3 | M6 | p value ** | Preop | M3 | M6 | p value ** | Preop | M3 | M6 | p value ** |  |
| Overall | 96.0 (27.9) | 89.8 (17.9) | 89.4 (15.2) | 0.43 | 55.2 (45.6) | 53.8 (43.4) | 48.9 (27.7) | 0.86 | 21.6 (12.3) | 36.2 (19.9) | 30.7 (18.3) | 0.03^#^ |  |
| Combined | 96.3 (28.5) | 89.8 (17.9) | 89.3 (15.2) | 0.13 | 50.2 (37.6) | 40.7 (29.3) | 47.5 (26.3) | 0.46 | 21.7 (10.6) | 37.6 (22.3) | 28.6 (14.6) | 0.09 |  |
| Standalone | 79.8 (46.5) | 93.8 (18.0) | 85.9 (6.9) | 0.56 | 44.7 (33.4) | 88.1 (8.3) | 52.9 (34.4) | 0.32 | 17.6 (15.6) | 32.5 (13.0) | 36.6 (27.4) | 0.17 |  |
| p value* | 0.63 | 0.81 | 0.35 |  | 0.90 | **0.04** | 0.71 |  | 0.48 | 0.80 | 0.61 |  |  |

|  | EMA | | | | DCD | | | |
| --- | --- | --- | --- | --- | --- | --- | --- | --- |
| Mean (SD) | Preop | M3 | M6 | p value ** | Preop | M3 | M6 | p value ** |
| Overall | 7281.0 (6087.7) | 25976.2 (30819.9) | 20228.6 (17858.1) | 0.01^##^ | 15.3 (11.0) | 24.9 (22.2) | 22.5 (18.1) | 0.26 |
| Combined | 8046.7 (6278.3) | 24653.3 (29498.1) | 20880.0 (17972.5) | 0.04^#*^ | 17.6 (15.8) | 20.3 (17.7) | 20.8 (19.6) | 0.80 |
| Standalone | 5320.0 (6295.1) | 35140.1 (37730.8) | 22320.0 (18832.6) | 0.22 ^#^ | 6.8 (5.5) | 36.6 (30.2) | 27.1 (13.6) | 0.09 |
| p value* | 0.26 | 0.63 | 0.90 |  | 0.20 | 0.21 | 0.20 |  |

Table 3. In Vivo Confocal Microscopy (IVCM) Study Findings during the follow-up. 3G. * Mann-Whitney U-test, comparing combined vs standalone procedures. DCD: dendritic cell density (cells/mm^2^); EMD: epithelial microcyst density (microcysts/mm^2^); EMA: epithelial microcyst area (µm^2^); GCD: goblet cell density (cells/mm^2^); M3: month3; M6: month 6; SMR: stromal meshwork reflectivity (arbitrary scale). **ANOVA for repeated measures. ^#^significantly higher when comparing the preoperative and the 3-month visit. ^##^ EMA was significantly higher in both postoperative visits
