# Supplementary material for: Longitudinal analysis of XEN45 gel stent bleb morphology using bleb grading scales, anterior segment-OCT, in vivo confocal microscopy, and impression cytology
Source: Graefes Arch Clin Exp Ophthalmol. 2025 Oct 3;264(1):207–18. doi: 10.1007/s00417-025-06952-0 (PMC12906558; doi:10.1007/s00417-025-06952-0)
Supplement: Supplementary file 14 — Supplementary Material 14 [file 417_2025_6952_MOESM14_ESM.docx]

|  | BECSD | | | BECSA | | | BSCSD | | | BSCSA | | |
| --- | --- | --- | --- | --- | --- | --- | --- | --- | --- | --- | --- | --- |
|  | M1 | M3 | M6 | M1 | M3 | M6 | M1 | M3 | M6 | M1 | M3 | M6 |
| Overall | 7.7 (5.3) | 5.7 (4.1) | 6.4 (4.6) | 46457.7 (35290.5) | 42753.4 (42464.3) | 64072.5 (61535.0) | 4.5 (4.6) | 3.9 (4.6) | 1.0 (2.1) | 218447.5 (351130.2) | 314304.0 (573405.4) | 202681.1 (445165.3) |
| Success | 8.2 (6.0) | 7.7 (2.5) | 8.2 (3.5) | 38521.4 (35081.7) | 57559.0 (41973.9) | 98717.1 (418781.7) | 4.2 (3.7) | 2.8 (4.1) | 0.7 (0.8) | 157909.7 (170856.6) | 496330.3 (742636.7) | 358698.1 (575436.5) |
| Failure | 8.1 (4.5) | 4.1 (3.6) | 4.0 (4.3) | 57568.6 (36252.9) | 24986.6 (39779.3) | 22499.0 (50309.2) | 5.0 (6.0) | 5.2 (5.3) | 1.4 (3.1) | 291092.9 (509555.9) | 95865.8 (152490.5) | 15460.8 (34571.4) |
| p value* | 0.65 | 0.17 | **0.03** | 0.64 | 0.30 | 0.30 | 0.26 | 0.05 | 0.15 | 0.93 | 0.70 | 0.29 |

(cont.)

|  | BH | | | BT | | | BET | | |
| --- | --- | --- | --- | --- | --- | --- | --- | --- | --- |
|  | M1 | M3 | M6 | M1 | M3 | M6 | M1 | M3 | M6 |
| Overall | 547.8 (132.8) | 558.1 (219.1) | 573.9 (287.4) | 231.6 (103.8) | 206.8 (83.1) | 226.0 (83.2) | 81.4 ()25.4 | 73.7 (16.2) | 73.1 (16.3) |
| Success | 569.9 (156.2) | 657.7 (249.4) | 707.7 (347.0) | 274.5 (125.1) | 203.4 (99.2) | 259.8 (101.2) | 88.0 (27.8) | 69.9 (16.2) | 75.0 (18.3) |
| Failure | 521.2 (109.4) | 438.5 (97.3) | 420.6 (45.7) | 205.6 (33.1) | 223.5 (86.2) | 196.9 (24.4) | 73.4 (22.5) | 78.3 (16.8) | 70.9 (15.2) |
| p value* | 0.72 | 0.10 | 0.11 | 0.04 | 0.72 | 0.27 | 0.20 | 0.58 | 0.72 |

Table 2. Anterior Segment OCT (AS-OCT) bleb quantitative measurements over time.

BECSA, bleb-wall epithelium cyst-like structure area (µm^2^); BECSD, bleb-wall epithelium cyst-like structure density (microcysts/image); BSCSA, bleb-wall sub-epithelium cyst-like structure area(µm^2^); BSCSD, bleb-wall sub-epithelium cyst-like structure density (microcysts/image); BH, Bleb Height (µm); BT, Bleb Thickness (µm); BET, Bleb Epithelial Thickness (µm); * Mann-Whitney U-test (success/failure comparison).
