# Supplementary material for: Longitudinal analysis of XEN45 gel stent bleb morphology using bleb grading scales, anterior segment-OCT, in vivo confocal microscopy, and impression cytology
Source: Graefes Arch Clin Exp Ophthalmol. 2025 Oct 3;264(1):207–18. doi: 10.1007/s00417-025-06952-0 (PMC12906558; doi:10.1007/s00417-025-06952-0)
Supplement: Supplementary file 15 — Supplementary Material 15 [file 417_2025_6952_MOESM15_ESM.docx]

|  | Baseline | W1 | M1 | M3 | M6 | p value** |
| --- | --- | --- | --- | --- | --- | --- |
| *XGS (all)*  IOP, mean (SD)  Meds, median (IQR) | 19.6 (4.0)  3 (2-3) | 10.6 (2.8)  0 (0) | 14.3 (2.2)  0 (0) | 18.7 (6.3)  0 (0-1) | 16.2 (4.9)  0 (0-1) | **<0.01^$^**  **<0.01** |
| *XGS (excluding XGS and DM2)*  IOP, mean (SD)  Meds, median (IQR) | 18.8 (3.4)  2.5 (1.5-3) | 10.2 (2.9)  0 (0) | 14.4 (2.0)  0 (0) | 20.8 (6.9)  0 (0-1.5) | 14.9 (3.1)  0 (0-2) | **<0.01^$$^**  **<0.01^$$^** |
| *p value**  *IOP*  *Meds* | 0.67  0.62 | 0.95  0.57 | 0.90  0.57 | 0.26  0.63 | 0.65  0.46 |  |

Supplementary Material 1: intraocular pressure (IOP) and medications (Meds) over time. IQR: interquartile range; SD: standard deviation; W: week; M: month. *Mann-Whitney U-test. ** ANOVA for repeated measures. **^$^** statistically significant except at M3; **^$$^** statistically significant except at M1
